# Identifying and investigating emergent behaviour in neural systems

**DOI:** 10.3389/fnins.2026.1752724

**Published:** 2026-07-24

**Authors:** Kyle C. A. Wedgwood, Patrick McGivern, Alexander R. Harris

**Affiliations:** 1 Living Systems Institute, Department of Mathematics and Statistics, University of Exeter, Exeter, United Kingdom; 2 School of Creative Arts and Humanities, University of Wollongong, Wollongong, NSW, Australia; 3 Department of Biomedical Engineering, University of Melbourne, Melbourne, VIC, Australia

**Keywords:** computational models, emergence, epilepsy, multiscale, reductionism, tissue models

## Abstract

The concept of emergence is key to understanding much of neurophysiology, neurodevelopment, and neurodegeneration. Many brain functions are underpinned by behaviour that emerges from interactions of components such as neurons, synapses, and glia. Perturbations such as ion channel mutations or trauma can lead to new types of, often detrimental, emergent behaviour. Typical approaches to neuroscience involve studying neural behaviour in a range of *in vitro, in vivo*, and *in silico* models designed to capture the key components of the brain region under investigation. Translating from these models back to the brain requires that the component interactions within the models can be mapped onto interactions in the brain. However, in many cases, this may not hold, leading to emergent behaviour that is not derivable from knowledge of the brain components in relative isolation. We thus need a framework that respects the prospective system-dependent differences in interactions. In this manuscript, we distil theoretical approaches to emergence into a practical framework for assessing system behaviour focussed on “novelty” as a necessary condition for emergence and identify a range of ways in which novelty can arise. We consider how different approaches for investigating system behaviour can be applied in different contexts and when these might fail. We provide examples of different forms of novelty in neuroscience contexts and then apply the framework to understanding the specific case of epilepsy. We end by outlining a set of guiding principles for designing experiments and models for understanding emergent behaviour in neural systems.

## 1 Introduction

The brain is one of the most complex organs in the body comprising ~90 billion neurons, thought to be the principal actors in information processing associated with sensory, muscular and cognitive function (Herculano-Houzel, 2012). The brain’s complexity has fascinated scientists for centuries and presents a central challenge for understanding how features ranging from memory, executive decision making, sensory processing, motor coordination, personality and consciousness are generated by base processes. Knowledge of how neural systems function also offers hope for treating currently intractable neurological diseases.

In many cases, neural systems are investigated via so-called “bottom-up” or “reductionistic” methods, particularly in experimental studies. Such methods attempt to derive neural system function by extrapolating from studies of individual neurons or individual types of neurons in relatively isolated environments that differ significantly from the complex environment of the brain. This typically involves patch clamping of individual cells, growth of homogeneous tissue cultures, or genetic manipulation techniques targeting a single gene. Using such studies to draw inferences about neural function within the complex environment of the brain can be problematic. For example, two-dimensional Alzheimer’s tissue models fail to display amyloid plaque formation found in three-dimensional models (Harris et al., 2020). Such cases involve behaviours that are often described as being *emergent*. Emergent behaviours raise questions about the relative limitations and advantages of different models and the connection between comparatively simplified experimental contexts and the much more complex environment of the brain.

The difficulty in addressing these issues is that there is no unified framework for identifying and investigating emergent features in neuroscience. Varying definitions of emergence may lead to misunderstanding or miscommunication of data and theories. Furthermore, a lack of experimental protocols for investigating emergent features biases research and theory towards reductionistic methods. This has led to growth of large experimental data sets but limited understanding of how this relates to system function, and often speculative data interpretation. Subsequently, this limits the ability to create and control system functions.

Our goal in this article is to establish a general framework for understanding emergence that can be applied to both experimental and theoretical neuroscience. We will do this by considering a variety of philosophical accounts of emergence and map these onto experimental and theoretical perspectives. Rather than advocating for a narrow definition of emergence, our goal is to clarify the core claims associated with emergence and the diverse ways in which those claims can be made more precise. Those variations can be associated with different contexts, with some accounts being more appropriate for theoretical contexts and others more appropriate for experimental contexts. As a consequence of this, our framework is agnostic towards standard categories such as ontological, epistemological, strong and weak emergence; we will discuss these concepts at various points throughout the manuscript, giving examples of how they relate to our framework. In Section 2, we describe different approaches that can be used to characterise systems. In Section 3, we discuss how behaviour in individual neurons and neuronal networks is typically categorised. In Section 4, we briefly review the historical literature on emergence. In Section 5, we present our framework for understanding emergence. In Sections 6 and 7, we apply this framework to some examples of neural systems, first by considering a range of applications to systems at different levels of biological organisation (Section 6) and next by considering the application of the framework to complex and clinically significant phenomena associated with epilepsy (Section 7). Finally, in Section 8, we provide a set of guidelines for the development of theoretical and biological models for gaining deeper knowledge of how brains function.

## 2 Strategies for describing systems

In this section, we describe five possible strategies for investigating neural system behaviour. In subsequent sections, we consider the relationship between these strategies and various accounts of emergence.

Our descriptions of all five strategies are idealisations of sorts: in practice, the investigation of system behaviour will often involve nuances and hybrid strategies that we ignore here. Describing the strategies in this way is useful for identifying and clarifying their core underlying assumptions about the nature of system behaviour.

We begin with a strategy we call Good Old-Fashioned Reductionism (GOFR^1^). GOFR represents an important strategy in its own right, and will also serve as a foil against which we will characterise four alternative strategies (Table 1). GOFR consists of three core principles: Derivability (concerning the relationship between system-level^2^ features and component features), Separability (concerning separability of system components), and Practical Optimism (concerning inherent challenges of understanding system-level brain function).

**Table 1:** Strategies for describing system behaviour.

System behaviour	Principle		
	Derivability	Separability	Practical optimism
GOFR	✓	✓	✓
Practical pessimism	✓	✓	–
Optimistic non-separability	✓	–	✓
Pessimistic non-separability	✓	–	–
NO-GOFR	–	–	–

### 2.1 Derivability

Every system-level brain function can be modelled and derived from the features and characteristics of a set of inter-related components (neurons, proteins, etc.).

Derivability represents the idea that system-level features can be understood in a bottom-up manner based on the features of the system’s components. This principle is quite general, and is consistent with a variety of distinct ways of understanding what constitutes a ‘derivation’, for example through formal deductions in propositional logic, or through explicit derivations in mathematical models. Derivability does not require that the same set of components and features are relevant for deriving every system-level brain function. Instead, different sets of components or features may be relevant for different system-level features. Derivability also does not require that components be restricted to those conventionally regarded as individual units, such as neurons or proteins. Instead, components could include complex units such as pairs of synaptically coupled neurons.

### 2.2 Separability

The component features and characteristics relevant for deriving system-level features can be adequately observed and understood by studying the components in relative isolation, as if they interacted in a “Lego-like” manner.

‘Relative isolation’ means the components need not be embedded in the normal brain context to be adequately understood, although they may need to be embedded in some particular context (i.e., maintaining cultured cells or brain slices). In some cases, these conditions may mimic the natural brain context, while other conditions may be deliberately unnatural to maintain a research standard, promote particular experimental responses, satisfy experimental constraints, or to facilitate comparison with existing datasets.

In its most extreme version, Separability treats components as completely non-interacting. Less extreme versions include interactions with the environment and/or other components^3^. In all cases, the relevant manner and characteristics of component interactions can be adequately observed and understood by studying the components in relative isolation.

### 2.3 Practical optimism

The third principle characterises a particular attitude toward the practical challenges involved in deriving system-level features. There are often practical challenges when deriving system-level features. There may be technical limitations in creating relevant, simplified models of a complex system. The relevant component features (including their normal relationships within the system—i.e., connectivity patterns) may be difficult to determine experimentally, making it difficult to create models with correct initial conditions. Derivations may also be extremely difficult since the number and variety of types of components may be large, and the inter-relations between components may be complex, requiring significant mathematical or computational resources to model system-level behaviour^4^. Moreover, the relevant timescales of the behaviours and interactions may differ significantly between the model of components and the system-level features of interest, again requiring significant computational resources.

Practical Optimism holds that although there may be significant practical challenges in deriving system-level features, we can expect these to be overcome through improved computational capability and measurement techniques.

### 2.4 Alternatives to GOFR

Since GOFR is based on three core principles, we can describe alternative strategies where different combinations of those individual principles are accepted or rejected. Also note, as GOFR is a claim regarding every system-level feature, its failure only requires a failure to explain some system-level features.

#### 2.4.1 Practical pessimism

The first alternative accepts Derivability and Separability but rejects Practical Optimism. On this view, system-level function is derivable ‘in principle’ from Lego-like component features, but the practical challenges are regarded as insurmountable (at least in some cases).

There are several possible motivations for Practical Pessimism. The computational requirements for deriving system-level brain function from its components may be enormous, because of the vast number of components and parameters involved and/or the short relative time-scales on which those components and parameters need to be modelled (and the much longer time-scales of system level brain function). A key part of this concern is that the complexity of brain activity cannot be simplified or ‘compressed’. Alternatively, the hurdles to adequate observation of the features and characteristics of the components are insurmountable, even when they are in relative isolation.

#### 2.4.2 Optimistic non-Separability

The second alternative maintains Derivability and Practical Optimism but rejects Separability. Here, system-level brain function can be derived from the features and characteristics of the brain’s components. These derivations may be extremely difficult, but we can expect those difficulties to be overcome (Practical Optimism). However, the relevant features and characteristics of components cannot be discovered by observing them in relative isolation.

A motivation for this view is that the component features and characteristics in relative isolation will be radically different from the natural context of the brain. Subsequently, extrapolation of information from the isolated context to the brain may be completely unreliable. However, more complex models that avoid treating the components as relatively isolated units may enable derivation of system-level function. These models may emphasize the role of other specialised contexts, such as artificial contexts that allow us to observe sets of interacting components (so, not in relative isolation), while still controlling and accessing those components in ways not possible in the brain, e.g., artificially created tissue structures, where the goal of modelling is not necessarily to match the structure of the brain, but instead to develop an understanding of the interactions between the relevant components and the rules and constraints that characterise them.

#### 2.4.3 Pessimistic non-Separability

A third alternative maintains Derivability while rejecting both Practical Optimism and Separability. The motivation for this view is the idea that Non-Separability should lead to greater pessimism about the possibility of being able to make adequate observations and models of the relevant components: if those components cannot be adequately studied in relative isolation, then the practical challenges associated with deriving system-level features from the features of components appear to be even more significant.

#### 2.4.4 No-GOFR

The final alternative is to reject GOFR entirely by rejecting Derivability, Separability and Practical Optimism. On this view, at least some system-level brain function cannot be derived from the features and characteristics of the brain’s components.^5^

### 2.5 Examples of different strategies describing systems in neuroscience

An example of the GOFR strategy in neuroscience showed that increasing seeding densities of mature primary rat cortical neurons led to linear increases in synapse densities (Cullen et al., 2010). Synaptically coupled neurons were treated as the components in a system represented by a two-dimensional neuronal network. Extrapolation suggests that synapse number can be inferred directly from knowledge of neuron number, so that complexity grows in a bounded and predicable way. A more controversial application of GOFR was the attempt by the Human Brain Project to mathematically model an entire brain by composing models of individual neuron and synapse behaviours (Markram, 2006). Less than a year from its start, this project was heavily criticised by the neuroscience community^6^ for being too narrow in its scientific focus, with areas associated with higher level behaviour, such as cognition, being essentially removed from the project in its second phase (Alfred, 2014). Criticism of GOFR includes the challenge of deriving system-level behaviours of even simple neural systems, such as the *C. elegans* nematode, which possesses only 302 neurons (387 neurons for the male) and whose neuronal network structure has been fully mapped (Cook et al., 2019).

Despite the criticism of GOFR, it is possible that many researchers are still drawn to GOFR (or something similar) because it seems to be the only optimistic attitude available, together with the thought that science should be driven by optimism (at least as a working hypothesis). However, there is another optimistic option available. An application of this Optimistic Non-Separability strategy appears in comparisons of two- and three-dimensional co-cultures of primary rat hippocampal neurons and astrocytes (Acero et al., 2023), in which axonal fascicles, neural polarisation and astrocyte domain formation appeared in three-dimensional, but not in two-dimensional tissue. Here, synaptically coupled neurons and astrocytes were treated as the components. The features observed in the three-dimensional tissue could not be derived from studies of isolated neurons or astrocytes, or even two-dimensional co-cultures. This shift in behaviour is mainly due to the cells in the two-dimensional cultures interacting with the culture plate and media, while in three dimensions, cell–cell interactions predominate. Subsequently, it may be possible to use the component features within the three-dimensional culture to derive features in more complex tissues. Thus, some features of three-dimensional tissue cannot be understood from relatively isolated components.

To our knowledge, the clearest contemporary example of the NO-GOFR strategy in neuroscience relates to some of the many accounts of consciousness, which is sometimes regarded as being inexplicable in compositional terms (Chalmers, 2008).

### 2.6 Connecting the five strategies with emergence

Since GOFR is designed to capture an extreme form of reductionism, and since emergent behaviours are often described as being ‘irreducible’ in some sense, our four alternatives to GOFR give us the beginning of a framework for categorising emergence. Rather than a simple opposition, where describing something as emergent means simply that it is ‘irreducible’ in some sense, attending to the three core principles of GOFR allows to distinguish between different alternatives to GOFR and hence different ways of understanding what emergence involves.

There is a long history of discussions of emergence with a variety of definitions. Accounts often begin by contrasting emergence and reduction and then distinguishing between ‘weak’ and ‘strong’ forms of emergence. In terms of the five strategies described above, GOFR represents the most strongly reductionistic, while NO-GOFR represents a type of strong emergence. Practical Pessimism represents the view most often associated with weak emergence, where derivations are ‘in principle’ possible. The two forms of Non-Separability (Optimistic and Pessimistic) represent stronger forms of emergence, with pessimism the stronger of the two claims.

Note that, of these five strategies, the two forms of Non-Separability have received relatively little attention in the literature. The primary reason for rejecting Separability is the thought that it does not adequately capture the manner in which interactions between components can vary across contexts, and the resulting differences between behaviours observed in relatively simple, isolated contexts and those found in much more complex ones. We suggest that this strategy is in fact the most promising one for understanding much of the brain’s behaviour, in particular because it highlights the fact that understanding neural systems may require studies of components in specialised contexts, rather than in relative isolation (Harris et al., 2020).

### 2.7 Summary

In this section, we have described five possible strategies for understanding neural systems and introduced the relationship between these strategies and the concept of emergence. Before describing this relationship with emergence in more detail (in sections 5 and 6), we first describe some of the key features of components and systems (section 3) and some historical concepts relevant to emergence (section 4).

## 3 Classifying neurons and neuronal systems

To determine the most appropriate strategy for assessing neural systems, we first investigate some key features of individual cells and neuronal systems typically highlighted as important for understanding the brain’s behaviour.

### 3.1 Individual neurons

Individual neurons are currently characterised by several attributes. One such attribute, initially studied by Cajal and Retzius focusses on neuronal morphology based on the number and distribution of axons and dendrites (Gil et al., 2014). Advanced imaging techniques are providing ever finer resolution of cell morphology (Vasques et al., 2016; Cervantes et al., 2019). Simultaneously, mathematical modelling of signal propagation and processing along neurites (McDougal et al., 2017; Allen Institute for Brain Science, 2004) are providing more information on the link between neuronal morphology and function. Note, however, that neuronal morphology is often dependent on interactions (e.g., synapse formation) with other neurons, making it challenging to study in isolation.

In response to stimuli, neurons ‘fire’ *action potentials* (*spikes*): depolarisations of their membrane that propagate along the nerve fibre. Action potentials are typically considered the base ‘unit’ of information in neural processing, encoding stimulus details. Temporal features, such as action potential frequency, are used to categorise neurons into ‘excitability classes’ (Hodgkin and Huxley, 1952). Certain neuronal classes display rhythmic ‘bursts’ of action potentials, interspersed by quiescent epochs enabling more reliable encoding of information (Lisman, 1997; Reinagel et al., 1999), and can be linked to expression of specific ion channels in the cell membrane (Jin et al., 2000; Swensen and Bean, 2003).

Voltage and patch clamp techniques (Verkhratsky et al., 2006; Verkhratsky and Parpura, 2014) enable neuronal classification based on ion channel gating properties, which drive electrical activity. This allows electrophysiological classification of neurons according to kinetics. The identification of abnormalities in specific ion channels (channelopathies), and their effect on action potentials have revised understanding of certain diseases and potential avenues for their treatment (Kim, 2014).

The most recent dimension of neuronal classifications applies ‘-omics’ sequencing capabilities, across multiple single-cell attributes. The transcriptome quantifies expression of mRNA, which serves as a blueprint for functional behaviour (Tang et al., 2009); the epigenome provides details about post-translational DNA modifications, which regulates gene expression (Bernstein et al., 2007); whilst the proteome captures information on protein expression, such as ion channels (Jiang et al., 2020). Other types of analysis such as lipidomics (Piomelli et al., 2007) and metabolomics (Qi et al., 2018) promise more ways to classify neuronal cells.

Techniques can also be combined for finer-grained classification. For example, a study comparing morphological and electrophysiological behaviour concurrently found neurons from the mouse visual cortex could be classified into 17 electrophysiological, 38 morphological and 46 morpho-electric types (accounting for the combination of morphological and electrophysiological properties; Gouwens et al., 2019). Similarly, the patch-seq approach combines patch clamp electrophysiology with single cell RNA (or other) sequencing to classify expression of specific genes with electrophysiological behaviour (Lipovsek et al., 2021).

### 3.2 Neuronal networks

Different forms of interaction exist between cells in neural systems, for example chemical (synaptic), electrical (gap junction) and ephaptic coupling. During development, individual neurons emit neurite projections that are precursors to axons and dendrites. If neurites from two neurons contact one another, a synapse may form, allowing direct communication between them. The precise mechanisms underlying synaptogenesis remain elusive, with cell adhesion molecules (Südhof, 2018; Batool et al., 2019), mechanical forces, and the internal cytoskeleton of each cell (Kilinc, 2018) playing a role. Neurons can also interact through ephaptic coupling, which does not involve direct contact between cells; and by changing the chemical composition of the local environment, affecting nearby cells.

**Figure 1:**
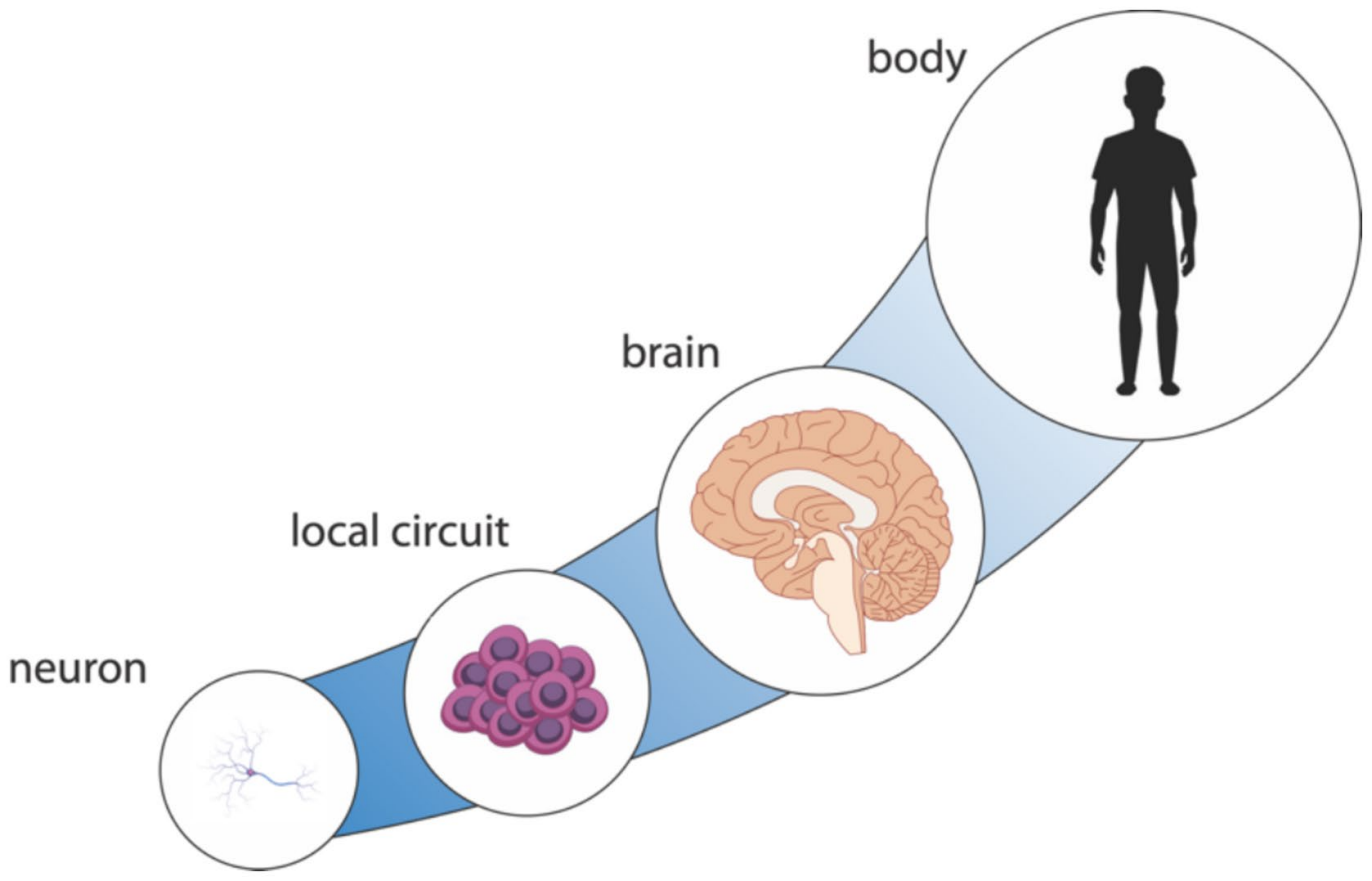
Neural system classifications at different scales.

Neural systems are typically classified as localised networks at small scales and regional networks at larger scales based on functional behaviour (Figure 1), in which they communicate with one another via synaptic and gap junction coupling (Silberberg et al., 2005; Hill et al., 2012; Yu et al., 2012). Neural networks often display rich dynamical behaviours that has often led them to be characterised as systems distinct from individual cells. For example, in the pre-Bötzinger complex, which regulates breathing patterns, near-synchronous bursting activity is reported during the inspiratory phase (Feldman and Del Negro, 2006; Kam et al., 2013). Spatially periodic spiking across grid cells in the entorhinal cortex is a crucial component of our navigation system (Hafting et al., 2005). In the hippocampus, sequences of activity encode paths in physical space, which can be used to consolidate spatial memory (Marozzi and Jeffery, 2012) and plan routes to remembered locations (Pfeiffer and Foster, 2013). At a higher level, verbal communication involves coordinated activity between auditory, language and speech processing areas, including the timing of activity, and coupling of oscillation amplitudes (Canolty and Knight, 2010). Neural systems can also be associated with pathological states: excessive synchrony in the basal ganglia is associated with rigidity and tremor in Parkinson’s disease (Oswal et al., 2016; Swann et al., 2015).

Furthermore, interactions between neurons and between brain regions dynamically change, particularly during development and learning. One popular way to understand such changes applies graph theory techniques to investigate the statistical similarity in activity between different regions. Regions with high statistical similarity are deemed *functionally connected*. Longitudinal analysis suggests that increases in *modularity,* that is, brain regions with dense connections within the region but sparse connections to other regions, are associated with learning in certain tasks (Bassett et al., 2011; Sporns and Betzel, 2016). The emergence of modular networks has also been postulated to explain facets of consciousness (Grindrod, 2018; Grossberg, 2017) and intelligence (Barbey, 2018). Importantly, this perspective focuses on the more abstract notion of how neurons and neuronal populations dynamically interact, rather than on specific neural properties.

### 3.3 Summary

There are different ways of characterising brain components and neuronal systems. Understanding the relationship between individual components and neural systems requires one of the five strategies we have described in the previous section. As we noted, four of those strategies can be associated with concepts of emergence. Hence, in order to understand the strategic options, we require a clearer account of emergence.

## 4 Historical and contemporary perspectives on emergence

The previous two sections outlined general, high-level strategies for investigating systems, components and emergence in neural systems. In this section, we review some different ways that emergence has been described across various philosophic and scientific fields. This review is not intended to be exhaustive but is supposed to serve two key purposes. The first is to introduce, in informal terms, some foundational concepts in emergence and contextualise them in neuroscience. The second is to highlight the diverse array of concepts and perspectives of emergence that could be applied to neural systems. We note that some of these concepts have typically been applied (or in some cases, developed) under distinct perspectives of the brain and neural systems, for example, with some considering the brain as a computational system, and others considering the role that embodiment of the mind plays in consciousness.

Emergence was cemented as a philosophical concept in the late 19th and early 20th century (Alexander, 1920; Broad, 1925; Lewes, 1874; Lloyd Morgan, 1923; Mill, 1843). Early incarnations primarily aimed to address the gap between a mechanistic perspective of the world, in which all processes, including life, can be understood solely by reduction to fundamental laws or forces; and a vitalistic viewpoint, which purports that living processes must differ from inert substances and hence cannot be reduced to the same fundamental laws. The emergentist perspective argued that a system can acquire properties distinct from its components. These ‘emergent’ properties were regarded as fundamental in the sense they cannot be understood by composition of component properties, but do not imply the more complex system differs entirely from its components [see O’Connor, 2021 for a general overview of the concept of emergent properties].

A key aspect of contemporary accounts of emergence involves the notion of novelty: networks of interacting components can support novel behaviours that differ from those of the individual components. For example, bursting activity of a cell in a network is novel if an isolated cell is unable to burst if taken out of the network (Smolen et al., 1993). Novelty may be realised as new computational abilities. For example, a neural network can perform sound localisation, whereas isolated neurons cannot (Grumiaux et al., 2022). A population of neurons in relative isolation might be able to discriminate sounds, but only when coupled to other populations that provide contextual information can speech and language be processed (Goldberg, 2016). At yet higher levels, interaction of language processing centres with frontal lobe and limbic system structures can impart emotional responses to dialogue (Nummenmaa et al., 2014).

As a concept, emergence can be divided into ontological and epistemological forms. In a broad sense, ontological emergence addresses true changes to the underlying system, whereas epistemological emergence only requires a change in how the system appears from a different perspective (McGivern and Rueger, 2010)^7^. In the context of neural dynamics, this is an important distinction since our understanding of macro- and microscopic phenomena is often constrained by the empirical tools available to measure them.

Emergent properties or behaviours typically depend on (or emerge from) the properties and behaviours of some underlying ‘base’ (often described as supervenience). Loosely speaking, a set of properties *B* supervenes on set *A* if no difference in *B* can occur without a difference in *A* also occurring (Lewis, 1986). For example, if we assume electrophysiological properties of a neural network supervene on those of the individual neurons, then we can only observe different firing activity at the tissue level if the firing activity of the cells also changes.^8^

Another common concept associated with emergence is ‘downward causation’ (Campbell, 1974), sometimes termed causal emergence, in which changes at the higher-level effect changes at the lower level. In neuroscience, this would claim that tissue behaviour affects individual cell behaviour. However, assessing claims about the direction of causation in these contexts can be difficult. For example, neural activity generates local field potentials. In turn, these potentials may modify the activity of individual neurons, through variation in local concentrations of ionic species. This appears to be a case of downward causation, since the product of collective neural activity—the local field potential—is exerting a causal influence on individual neurons. However, at the same time, the individual neurons are responsible for generating the field potential, suggesting that they should be understood as the proper causal agents engaging in ‘sideways’ interactions with one another rather than being influenced by some higher-level cause.^9^

Given the above, one set of constraints to describe a property as being emergent (O’Connor, 1994) requires that it supervenes on its lower-level substrates as well as exhibits downward causation on them, whilst possessing properties not present in them. Other, weaker instantiations of emergence instead focus on the predictability of the emergent features. For example, one account defines a property as weakly emergent if it can be derived from the properties of its components only through simulation which cannot be compressed to a simpler model (Humphreys, 2008; Bedau, 1997, 2011). In a practical sense, the notion of incompressibility is hard to demonstrate, limiting its utility.

Emergence is often explored in the context of complex systems, typically in networks of large numbers of similar interacting components [including cases where the number of components is treated as infinitely large (Anderson, 1972)], or in smaller networks of heterogeneous components. However, we remark that complexity is neither sufficient nor necessary for emergence.^10^ Indeed, simple systems including the undamped pendulum can display strong forms of emergence (McGivern and Rueger, 2010), and coupled networks of large numbers of cells may display uncoordinated behaviour akin to their uncoupled dynamics, so that no emergent features are evident.

From a computational perspective, there are two broad approaches that consider to what extent higher-level system behaviour decouples from lower-level behaviour. One approach considers the extent to which the higher-level systems are closed with respect to information theoretic measures, such as entropy. Briefly, if a system has a higher level (often defined through coarse-graining) for which knowledge of lower-level features does not offer any reduction in uncertainty, then the higher-level system can be thought of as decoupled from the lower one and hence emergent. This approach has been adapted and applied in a variety of settings under various names including information theoretic emergence (Mediano et al., 2022; Rosas et al., 2024), causal emergence (Hoel et al., 2013; Rosas et al., 2020; Klein and Hoel, 2020; Hoel, 2025), coarse-graining (Flack, 2017; Kolchinsky and Wolpert, 2018; Barnett and Seth, 2023) and has been used to coarse-grain simulations of models from the Virtual Brain Project (Milinkovic et al., 2025). Alternatively, one may consider what behaviours occur as system sizes grow or shrink (Butterfield, 2011). These computational approaches provide a concrete way of examining of emergence with respect to the five identified strategies that may be applied to neural systems. However, we note that they require computational models to be formulated,^11^ which may not be appropriate to study every way in which a neural system might exhibit emergence, and may only describe emergence in the model rather than in the neural system itself.^12^

We end this section by remarking that there are several other contemporary perspectives on emergence which involve characteristics leaning more towards theoretical neuroscience, such as Wilson’s *degree of freedom* viewpoint^13^ (Wilson, 2010), or towards experimental neuroscience such as Humphreys’ notion of *fusion*^14^ (Humphreys, 1996, 1997). Finally, we remark that emergent systems are sometimes described as self-organised. Self-organisation implies that the components arrange themselves into a complex structure and does not require a “guiding hand.” We acknowledge that there are significant overlaps between self-organisation and emergence, but do not consider them to be equivalent.

### 4.1 Summary

This section highlights the diversity of approaches for considering emergence and how they might be applied in a neuroscience setting, noting that different approaches may have distinct baseline assumptions about which neural features are important. The variety of definitions and ‘starting points’ often makes direct comparison between different approaches challenging, particularly, in a field as broad and interdisciplinary as neuroscience. Hence there is a need for a broader, unifying framework that is applicable to many of the above approaches, and clarifies their interconnections.

## 5 A revised perspective on emergence for neuroscience

Here, we aim to establish a framework that captures the diverse ways in which the concept of emergence can be applied in both experimental and theoretical neuroscience. Importantly, framework concepts should be interpreted in the same way in both sub-disciplines. Furthermore, the framework should be applicable to all scientific disciplines, enabling a uniform understanding of emergent phenomena in theoretical models and experimental systems.

Our framework identifies some essential features of emergence (Tables 2, 3) and some non-essential features (Table 4). Importantly, we eschew complexity and downward causation as essential concepts for defining emergence in neuroscience and instead focus on two scenarios leading to emergent behaviours. In the first, **a system of interacting components acquires features that are not present in the individual components**. In the second, **individual components exhibit novel behaviour as a result of being part of a system**. Note that in the second scenario, we are not necessarily interested in the dynamic behaviour of the system as a whole; the key point is that novel behaviour emerges, regardless of whether it occurs in the individual components or the system.^15^

**Table 2:** Framework for assigning emergent phenomena.

Feature	Description
Collective	Emergent behaviours arise in systems of interacting components (‘collectives’).
Dependent	Emergent behaviours of collectives are generated by and depend on the behaviours of the components.
Collaborative	Emergent behaviours arise through the collaborative interactions of the collective. They are not controlled by or located within particular individual components.
Novel	Emergent behaviours are in some sense novel relative to the features or behaviours of the components.

**Table 3:** Potential forms of novelty in collective systems.

Form of Novelty	Description
Definability	It may not be possible to define emergent behaviours in the same manner as the behaviours of the components.
Behavioural complexity	Emergent behaviours of a system may be more or less complex than the behaviour of its components.
Locality	The expression of emergent phenomena may be uniform or localised in spatial or temporal regions of a collective system.
Measurability	It may not be possible to measure emergent behaviours in the same manner as the behaviours of the components.
Predictability	Emergent behaviours may not be predictable from the behaviours of the components.
Irreducibility	The behaviour of a collective system may not be reduced to a simpler representation of its emergent phenomena.

**Table 4:** Non-essential features of emergence.

Concept	Description
System complexity, diversity and scale	Emergent behaviours are often associated with degrees of complexity, diversity and scale in a system. However, this is not a universal requirement: emergent behaviours can be found in relatively simple and homogeneous systems, and the behaviours can occur on spatial and temporal scales that do not significantly differ from those characterising the system’s components.
Context dependence	Emergent behaviours are often context dependent, since the surrounding environment can affect the behaviours of the components.
Model simplicity	Emergent behaviours can often be modelled using simplified systems. However, in some cases, models of emergence must incorporate a high level of detail to be successful.
Multilevel	Emergent behaviours can be multi-level, where interactions between emergent phenomena give rise to higher-level emergent behaviours.
Universality	The same generic type of emergent behaviour can be realised in different ways within different systems, irrespective of the lower-level system properties. These generic behaviours often conform to general higher-level laws that are described as being universal.
Upward and downward causation	In some cases, emergent behaviours can result in ‘upward’ and ‘downward’ changes across different levels in a system. However, this is not a necessary feature of emergence and identifying cases of upward and downward causation is tangential to the identification of emergence itself.
Nonlinearity	Emergent behaviours cannot be treated as linear products or compositions of lower-level behaviour.
Open/closed loop	Interactions between components can be uni- or bi-directional

### 5.1 Features of emergence relevant for neuroscience

The essential features required under our framework for a phenomenon/behaviour to be emergent are summarised in Table 2. Emergent phenomena involve systems of interacting components, where those interactions collaboratively generate novel behaviours. Each of the four features (Collectivity, Dependency, Collaboration, and Novelty) requires further elucidation. The point of beginning in such general terms is to highlight the fact that the concept of emergence involves a cluster of distinct ideas that can each be made more precise in various ways, thus generating different ‘species’ of emergence.

We note the distinction between the terms ‘collective’ and ‘collaborative’ and remark that both are required. It is not enough for a system to simply possess several components; there is a further requirement that interactions between components are what produce the emergent behaviour. Interactions may be direct, such as the emission of chemicals, energy, charge or information; or indirect, by modifying the local environment. Interactions must be collaborative, in contrast to the existence of specific components that act as orchestrators of collective behaviour. For example, consider a Mexican wave in a sports stadium, no one individual leads the wave, as waves may be initiated and terminated at any point (Farkas and Vicsek, 2006). By contrast, Ca^2+^ waves in the heart leading to muscle contraction are initiated at a single location (the sinoatrial node) acting as a pacemaker (Lakatta et al., 2010).

Application of our framework requires clear understanding of neuroscience-specific components, systems, interactions and the concept of novelty, which will be expanded on in the following sections.

Note that our framework encompasses both epistemological and ontological conceptions of emergence. In particular, different forms of novelty (described below) can be understood as having epistemological or ontological bases, depending on whether novelty is associated with how a system’s properties and behaviour appear from a particular theoretical or observational perspective (epistemological), or whether novelty is associated with the properties and behaviour of the system itself (ontological).

### 5.2 Neuroscience-specific systems, components, and interactions

Emergent phenomena are often defined by the behaviour of a system or its boundaries. In the context of neuroscience, behaviour is often categorised by various measurement techniques, such as electrical recordings or magnetic resonance imaging (MRI). The boundaries of the system are often defined spatially, such as total brain structure, entire nervous system or body. This system may be classified as a single layer, or by the properties or interactions of its constituent components. To achieve this, we must be able to clearly identify the units that comprise the lower and higher-level systems, their properties, boundaries and interactions. From a neuroscience perspective, it is tempting to define cells such as neurons and astrocytes to be the lower-level units and tissue such as cortical microcircuits to be a higher level. However, we should remember that cells themselves are complex systems composed of building blocks such as RNA, proteins and lipids. In the opposite direction, microcircuits are organised into larger functional units which may also possess emergent properties.

The definition of components in neuroscience could therefore be taken as individual ions, certain molecules, biochemical pathways, organelles, cells, microcircuits or others. The interactions between components in neuroscience are often simplified to electrical fields but may also be chemical, pH, temperature or mechanical forces. In general, the strength and robustness of these interactions varies across scale (for example, compare the formation of an atom from its subatomic particles, neural network from its composing cells, and social interactions between people^16^). The interactions can have spatial and temporal characteristics, such as a neural structure and spike timing. Key to this approach is determining which components or interactions result in the emergent phenomena of interest. The choice of components, systems, boundaries and properties is often context dependent and may be limited by the tools available. Furthermore, interactions considered normal under typical conditions may not occur in atypical conditions. For example, under/over-packing seeding density of neurons on microelectrode arrays (MEAs) can lead to significant differences in synaptic communication.^17^

One must also consider the role of the external environment, which may contribute to, or suppress, emergent behaviour. Biological experiments are typically performed in tightly controlled conditions. One can therefore only study emergence within the confines defined by that environment. For *in vitro* experiments, this is further exacerbated by the steps required to maintain the tissue. For example, factors such as ion concentration and growth factors are often regulated in tissue cultures, but may generate emergent behaviours not observed in normal conditions. One potential avenue to avoid this issue is to regard the environment as being a component of the system, rather than being external to it. In this respect, theoretical models can be used to derive hybrid experimental systems that allow for the interaction of biological tissue and external environments to be studied more systematically.^18^

### 5.3 Representations of novelty

The notion of ‘novelty’ captures the idea that emergent behaviours in the collective system are somehow different than the behaviours of the components taken in relative isolation. Novelty can be characterised more precisely in various ways, giving rise to different varieties of emergence. In Table 3, we summarise some forms and aspects of novelty in collective systems; other forms of novelty may also be possible.

Each form of novelty can be illustrated in a neuroscientific context. Here we provide a series of examples for each form individually. In the subsequent two sections we will describe increasingly complex neuroscientific examples that can exhibit multiple forms of novelty at the same time.

#### 5.3.1 Definability

It may not be possible to define emergent behaviours in the same manner as the behaviours of the components. Features and properties of systems at coarse scales may not be definable at finer scales.

*Example:* Electrophysiological behaviour in single cells involves categorising different ionic currents by the dominant direction of current flow (inward vs. outward) and whether the current is inactivating. At the network level, electrophysiological behaviour is typically defined via neuronal firing rates or local field potentials representing an average of the synaptic currents. Here behaviours are typically categorised by synchronicity of spiking events, or oscillatory power within specific frequency bands. This distinction is equally valid for theoretical models. Single cells are often described via Hodgkin-Huxley formalism (Hodgkin and Huxley, 1952), which models ionic currents. By contrast, neural mass models describing neural populations (e.g., H. R. Wilson and Cowan, 1973) disregard ionic currents and instead describe firing rate dynamics. In general, there is no explicit mathematical mapping between these two levels of definition.

#### 5.3.2 Behavioural complexity

Emergent behaviours of a system may be more or less complex than the behaviour of its components.^19^

*Example:* The emergence of more complex system behaviour in seemingly simple neuroscience systems have been documented in models of autoassociative memory formation (Hopfield, 1982). Emergence of higher complexity behaviour can also occur as the network size or coupling strength between neurons changes. For example, consider a network of isotropically-coupled homogeneous neurons exhibiting synchronous electrical oscillations. As the coupling strength between neurons is varied, this synchronised state may give way to a *weak chimera* state in which neurons become grouped into two or more subpopulations (Bick and Ashwin, 2016). The neurons in each subpopulation remain synchronised, but the subpopulations now oscillate at different frequencies to each other so overall network rhythm becomes more temporally complex.

*Example:* A reduction in behavioural complexity can be observed in epileptic seizures associated with hyper-synchronous oscillatory neural activity (in the normal state, electrical activity scales approximately linearly with number of neurons in the system). In fully synchronous oscillatory behaviour, the entire network can be described by a single dynamic variable representing the phase of oscillation. Similarly, certain mathematical models of infinitely large neuronal networks can be exactly mapped to just two equations for mean voltage and mean firing rate (Montbrió et al., 2015).

#### 5.3.3 Locality

The expression of emergent phenomena may be uniform or localised in spatial or temporal regions of a collective system. The system may shift from exhibiting uniform behaviour to behaviour that differs depending on where it is observed (or vice versa) in contrast to the uniformity of its components.^20^

*Example:* During focal seizures, electrical activity transitions from a state typically involving low levels of synchrony across relatively uniform neural composition, into a state of localised hyperactivity and synchronicity. This hyperactive behaviour does not spread across the whole tissue. This localisation is not necessarily dependent on special properties of the tissue in the focal region (Junges et al., 2020) [and so surgical removal of epileptogenic zones does not always lead to seizure reduction (Goodfellow et al., 2016)]. More importantly, the localisation of electrical activity at the seizure site is dependent on the state and structure of the whole brain network so that the system cannot be decomposed as a collection of smaller, non-interacting subsystems.

*Example:* Localisation of activity can occur in mathematical models where, under regional perturbation, a disordered firing state can be replaced by a chimera state, which exhibits regions of localised, coherent dynamics surrounded by regions of incoherent, chaotic dynamics (Abrams and Strogatz, 2004). This type of behaviour, known as a bump attractor, has been used as an analogy for working memory (Avitabile et al., 2023).

#### 5.3.4 Measurability

It may not be possible to measure emergent behaviours in the same manner as the behaviours of the components.

*Example:* A cell’s phenotype depends on genetic and environmental factors and may be regarded as an emergent property. The methods used to measure the cells genetic and phenotypic features must differ. For example, genetic sequencing can determine the type of Na^+^ ion channel expressed in a cell, while electrical hyperexcitability of that same cell must be measured via electrophysiological experiments.

*Example:* Which “measurements” are used to analyse behaviour in mathematical models is dependent on the context and scale of the behaviour the model is attempting to describe. For example, Markov chain models can be used to describe transitions between conducting and non-conducting states of ion channels (Wedgwood et al., 2018). Here, the measurement is the number of channels that are in the conducting state at any given time. Increases in the number of a specific type of ion channel would thus lead to an increase in the system size. When describing electrical activity in whole neurons, it is often more useful to use a Hodgkin-Huxley model formalism (Hodgkin and Huxley, 1952) that foregoes describing the states of the channel themselves and instead defines a measurable variable that describes the proportion of channels that are in the conducting state. In this framework, increases in Na^+^ channel expression are reflected by increases in the conductance parameter for the Na^+^ current, with no associated change in system size.

#### 5.3.5 Predictability

Emergent behaviours may not be predictable from the behaviours of the components. There are several notions of predictability associated with emergence. In our exposition, we are not interested in quantitative prediction of emergent behaviour once it is known that emergent behaviour exists (as might be done via statistical modelling, for example). Instead, we focus on the predictability of a system’s behaviour given the known properties of the relatively isolated system components. This notion of predictability can be extended to four linked questions:

1. Are a system’s behaviours predictable from studying the components in relative isolation?
2. Supposing we can predict some form of novel behaviour will occur in a system; can we predict what type of behaviour this will be?
3. Supposing we can predict novel behaviour of a system in one condition; can we predict behaviour of the same system in other, distinct conditions?
4. Supposing we can predict novel behaviour in a system; can we predict how this behaviour will change upon modification of the system’s components?

Question 1 asks whether the systems behaviour can be fully predicted from known properties and interaction rules of relatively isolated components.

*Example:* A single neuron in media would express various ion-channels and pores in its membrane enabling it to interact with the environment, giving the impression that two neurons could only interact indirectly via changes to the local environment. Only by growing two neurons together in media would the formation of a synapse, allowing direct communication between the neurons, be observed. Therefore, a synaptically coupled neural system would not be predictable from individual neurons in isolation.

In question 2, it is assumed that the system will exhibit emergent behaviour, but it is not clear exactly what this will be.

*Example:* Mutations of the sodium ion channel SCN1A, which are associated with epilepsy, are known to affect channel function and network activity (Goldin and Escayg, 2010). However, it is not *a priori* clear whether such mutations lead to increases or decreases in overall network activity. Hence, whilst changes to tissue activity could be predicted by examining transcriptomic changes, the type of behaviour observed could not. In mathematical models, bifurcation theory predicts where we might expect to observe different kinds of behaviour, such as seizure onset (Jirsa et al., 2014). However, this analysis typically only provides predictions at small times following passage through a bifurcation; the observed system behaviour at larger times may be significantly different than that predicted by the (local) bifurcation theory.

Question 3 asks whether the behaviour of a system in a given condition can be predicted from knowledge of the same system’s behaviour in other conditions (e.g., altered environmental temperature or pH).

*Example:* Dravet’s syndrome is a severe form of epilepsy often associated with SCN1A mutations. These mutations result in an increased propensity for seizure initiation in elevated temperatures, for example, during high fever, so that the behaviour of the brain changes significantly under temperature variation. Moreover, the prevalence of associated temperature-induced seizures decreases with patient age, suggesting that longer-term physiological changes affect how the system behaves in different environments.

Question 4 asks whether changing properties of the components of a system would change the emergent behaviour.

*Example:* Parkinson disease is associated with a loss of dopaminergic neurons in the basal ganglia. This change in system composition leads to the emergence of excessive synchrony in neural activity within this region, which is associated with rigidity, movement difficulty, and tremor (McGregor and Nelson, 2019). One potential approach to treating this disease, is to implant hiPSC-derived dopaminergic neurons to replace those that have been lost (Harris et al., 2022a). However, it is unclear, particularly over the longer term, how the overall network, composed of native and implanted neurons, would behave (especially as the network itself adapts to the introduction of new cells). In mathematical models of synaptic-coupled neuronal dynamics, increased synaptic coupling strength can lead to catastrophic changes in overall network behaviour (Ermentrout and Kopell, 1990).

#### 5.3.6 Irreducibility

The behaviour of a collective system may not be reduced to a simpler representation of its emergent phenomena. Reducibility involves describing behaviour in terms built upon known laws and relationships. This can be viewed in two ways. One *bottom-up* approach addresses whether behaviour at higher levels can be deduced from knowledge of lower-level behaviours. This may be applied following an “in principle” fashion whereby a high-level system is irreducible if it is impossible to derive a description of its higher-level behaviours from a description of its lower-level ones. It may also be applied in a more practical sense whereby deriving higher level behaviours from lower-level behaviours is either infeasible due to the complexity involved in doing so or does not yield any greater understanding than describing the system at the higher level directly.

*Example:* It is not clear to what extent equations describing action potential dynamics could be derived from those describing the movement of individual ions. Similarly, even if the motion of such ions could be measured, it is not clear that this would allow us to predict action potential dynamics.

Irreducibility can also be considered from a *top-down* perspective, which addresses whether the higher-level behaviour could be observed in any simpler or smaller system comprising the constituent system components.

*Example:* A neuron contains a vast number of different chemical species, but the majority have no impact on action potential dynamics and can be ignored when studying this property. This can be described as dimensionality reduction or robustness of an emergent feature to a range of chemical compositions. From a mathematical standpoint, behaviour near dynamical state transitions can be understood through bifurcation analysis, which involves transforming the original model to a minimal model that captures the core features of the transition. The transformed model is irreducible in the sense that it is the smallest description that faithfully reproduces the qualitative dynamics of the original system. The analysis is useful for comparing different types of emergent behaviour, for example, by grouping neurons into different excitability types based on the bifurcations that lead to action potential generation.

### 5.4 Non-essential features of emergence

There are a range of other concepts traditionally associated with emergence (Table 4). In our framework, we consider these concepts non-essential, either because they do not help in understanding emergence or because they are associated with specific scenarios of emergence, rather than describing a common or universal theme. However, these concepts may still be relevant for describing characteristics of emergent behaviour in specific contexts.

### 5.5 Summary

Emergence is primarily associated with novel behaviour or novel properties of a system composed of lower-level substrates, as defined in Table 2. The novelty associated with the system behaviour in relation to the relatively isolated components may take many forms and possess many properties as presented in Table 3. There are additionally several properties that are associated with, but not essential for emergence, as described in Table 4. Importantly, there is no prerequisite for either the lower-level or higher-level system to be complex, but the emergent phenomena must be such that they are not reducible to any composition of properties of the lower-level components. Presently, we leave free a prescription of what the lower-level and higher-level components should be, instead remarking that emergence can be present at any number of intermediate levels within neural systems and is not restricted to biological systems.

## 6 Applying our emergence framework to simple neuroscience systems

**Figure 2:**
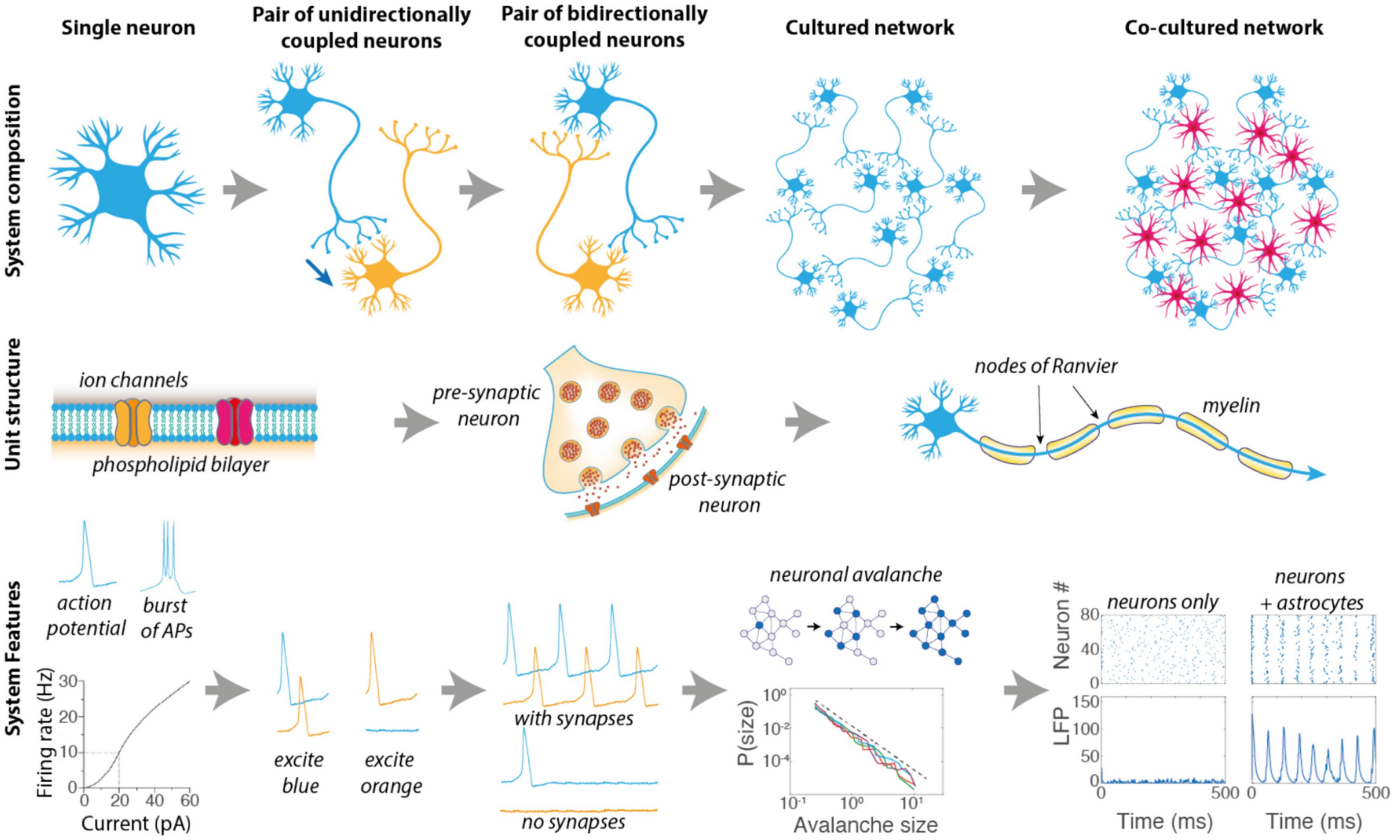
Examples of components in neuronal networks and emergent behaviour at different scales. *Left:* Action potentials in a single neuron arise due to the dynamic interaction of ion channel proteins that facilitate passage of charged ions across the cell membrane. Depending on the dynamic behaviour of these proteins, the action potentials may be isolated in time or may be grouped together as a burst. *Middle:* When cultured together, neurons form neurites that seek out other neurons and facilitate the formation of synapses. Once formed, synapses imbue a network of neurons with a sense of direction in terms of a ‘flow’ of electrical activity. This can lead, for example, to the formation of ‘half centre’ oscillators in which a network of synaptically-coupled neurons exhibits robust oscillatory activity even though each individual neuron does not exhibit oscillatory activity when decoupled from the network. In larger networks, the flow of electrical activity can be realised as a ‘neuronal avalanche’ in which excitation of a single neuron can ultimately spread across the entirety of the network. *Right*: Glial cells, such as oligodendrocytes and astrocytes, provide a range of support for neurons, including the formation of electrically insulating myelin sheaths which vastly increase the conduction velocity of action potentials along axons. Combined cultures of neurons and oligodendrocytes exhibit globally synchronised electrical oscillations, known as ‘population bursts’, which are not present in networks cultured from neurons alone. The population bursts are thus an emergent property of the co-cultured network.

Using simple neuroscience examples, we will now apply our framework to demonstrate identification of emergent behaviours without specifiying specific strategies for each case. Figure 2 displays illustrative examples of emergent electrical behaviour in neuronal networks, with system complexity increasing from left to right. The top row indicates example neuronal system compositions; the middle row schematises some of their important components; whilst the bottom row depicts typical features of their electrical behaviour.

To begin, the leftmost column depicts a single, isolated neuron which, under sufficiently large stimulation, exhibits action potentials. Action potentials are generated by the movement of ions, facilitated by ion channels, across the neuron’s membrane. The opening and closing of the ion channels are highly dynamic nonlinear processes, with the action potential considered as emerging from the interaction of ion channels via chemical gradients/electric fields embedded in an aqueous environment. This system provides an example of Definability as a form of novelty, with the ‘state’ of an isolated ion channel being defined via its conducting (open) and non-conducting (closed/inactivated) conformations. Conversely, the electrical state of the cell membrane is typically defined via the potential difference across it. Whilst the probability of ion channels being in each state is dependent on the voltage across the cell membrane, the ion channels themselves cannot define this voltage. Measurability is also evident as a form of novelty with the voltage or current across the cell membrane measured via standard electrophysiological techniques, while the instantaneous state of a single channel is inferred indirectly via conductance changes. Novel forms of Complexity can also be observed in the characteristics of action potential timing. Depending on the types and number of ion channels, action potentials may occur as isolated temporal events or as a burst. Bursting behaviour typically arises due to the presence of ion channels with slow rates of opening or closing. Hence, bursting action potentials can emerge when the composition of ion channels within a neuron changes, as observed in neuroendocrine cells under modulation of specific types of ion channel(s). These experiments show that a particular type of Ca^2+^-sensitive K^+^ channel plays a significant role in establishing bursting activity (Tabak et al., 2011). However, knowledge of the kinetics of this channel alone would not be sufficient to establish whether it would burst, since bursting would also depend on Ca^2+^ handling in other regions in the cell. Hence this is an example of lack of Predictability.

The second to left top panel depicts co-cultured neurons which develop neurites, enabling the formation of synapses for signal transmission between them. The formation of synapses involves the aggregation of proteins, such as ion channels, on and near the membranes of both neurons. This aggregation is typically asymmetric across the synapse, with *presynaptic densities* involving mechanisms for neurotransmitter secretion forming in one cell, and *postsynaptic densities* containing many receptors that respond to such neurotransmitters forming in the other cell. This structural asymmetry thus imposes a definition of one neuron as being *pre-synaptic* (blue) with the other designated as being *post-synaptic* (orange). It also indicates a direction to the flow of electrical information in the neural network. This acquisition of a new directional attribute (or symmetry breakdown) is an example of a change in Definability for the system, whereas isolated cells have no notion of direction. This has significant consequences for the cell, since the neurites ultimately become axons and dendrites, which have significantly different morphological and electrical properties. Note, however, that this definition applies on a synapse-by-synapse basis. A neuron could be a pre-synaptic for one synapse but post-synaptic for a different synapse.

Illustrated in the top-middle panel is an example of a ‘half centre’ oscillator (Marder and Bucher, 2001); a neuronal system consisting of two bidirectionally coupled neurons in which electrical oscillations at the network level emerge due to synaptic activity. In isolation, these neurons do not exhibit any sustained electrical oscillations. With synaptic coupling intact, the two-neuron network establishes a rhythm in which each neuron spikes 180° out of phase with the other. If any of the synaptic interactions are removed, the network does not exhibit oscillations. Hence, the half-centre oscillator exhibits Irreducibility and the rhythms it produces may be considered to be emergent. Half-centre oscillators can be formed in networks with either excitatory or inhibitory coupling, with the latter type involving more intricate interactions with intrinsic (i.e., non-synaptic) neuronal properties. Similar scenarios with a range of different phase-relationships are possible in networks with more neurons and, collectively, these networks are associated with so-called *central pattern generators* that underpin many functions, such as locomotion and gastration (Marder and Bucher, 2001; Prinz et al., 2004).

In larger networks, as illustrated in the second to right top panel, electrical activity can propagate rapidly in a wave-like manner. This represents an example of Locality since the states of neurons in different areas may exhibit markedly different behaviours, both in terms of single cell behaviour (e.g., firing rate) and in terms of behaviour relative to the surrounding tissue (e.g., degree of synchronicity). Strikingly, such propagation can often be instigated by the excitation of a single neuron due to the random opening and/or closing of its ion channels. Epochs of this type of spreading activity are known as *neuronal avalanches* and are typically characterised by the overall number of neurons that become excited before the network returns to its resting state (Pasquale et al., 2008). One frequent observation in neuronal networks is that the size distribution of avalanches is *scale-free* in the sense that it scales exactly in proportion to the number of neurons in the network. As such, although they appear temporally complex, at coarse scales the avalanches represent behaviour of a lower degree of Complexity than the full system possesses. That this property is preserved across networks of a range of sizes suggests the formation of avalanches is an emergent property of the network.

The far right panel depicts the interaction of glial cells, such as oligodendrocytes and astrocytes, with neurons. In some cases, this can lead to the formation of myelin sheaths, which vastly increase the conduction velocity of action potentials along axons. This provides an example of loss of Predictability since it is not possible to predict myelin formation or behaviour given knowledge of neurons and glial cells in isolation from one another. Other forms of co-cultures result in globally synchronised electrical oscillations, known as ‘population bursts’, which are not present in cultures of either, individual cell type (Alsaqati et al., 2020). Moreover, bursts appear after several days in culture and their presence is taken as a measure of the cultured network’s maturity. Similar transitions have been observed in which cultured networks transition from quiescence through disorganised firing patterns to synchronised population bursting under variation of the extracellular Ca^2+^ (Penn et al., 2016). The bursts, which often exhibit a high degree of periodicity, represent a lower degree of behavioural Complexity than that of which the whole network is capable. Finally, since almost all the neurons participate in the population bursts, the bursts represent an example of Locality, in the sense that the uniform bursting contrasts with the spatially disordered, incoherent firing in the absence of glia. Hence, the introduction of glia induces a transition from localised neuronal activity in networks without glia, to synchronised (i.e., temporally localised) bursting in networks with glia. The population bursts are thus an emergent property of the co-cultured network.

## 7 Applying our emergence framework to a complex neuroscience system

In this section, we will explore emergence in the specific context of epilepsy, which displays a range of emergent characteristics. In this context, epileptic seizures are a system property, while the components are undefined but are typically considered to be neurons. Extension of this examination could be made to other brain features and behaviours which change over time or due to different biological processes. It may be applied to development and learning, which involve the formation of new cells, cell types, and connections leading to many emergent features. Conversely, disease and aging can alter or lead to a loss of cells and connections and thus also lead to the emergence or loss of novel features and behaviours.^21^

Epilepsy is a disorder which is diagnosed phenotypically through abnormal brain activity and seizures (Fisher et al., 2014). Monogenic abnormalities account for 1–2% of cases, with the vast majority being polygenic, caused by environmental factors or trauma. Seizures may be provoked (e.g., by strobe lights) or unprovoked, and have a focal or general onset. Individuals undergoing a seizure may or may not have awareness and motor symptoms.

The International League Against Epilepsy have recommended 3 definitions of epilepsy (Fisher et al., 2014). An individual has epilepsy if they have: (1) at least two unprovoked or reflex seizures more than 24 h apart; (2) one unprovoked or provoked seizure with probability of further seizures similar to the general recurrence risk (at least 60%) after two unprovoked seizures, occurring over the next 10 years; (3) diagnosis of an epilepsy syndrome. The condition is considered resolved if the patient has suffered no further seizures after 10 years and is off antiseizure medications for 5 years. These definitions aim to align with how physicians and patients think about epilepsy and assist with treatment decisions. However, they present issues around quantification of seizure risk and defining what an epilepsy syndrome is. Seizure risk is determined from population studies including measurement of abnormal electroencephalogram (EEG) after a first seizure which subsequently correlates with a second seizure. However, an individual’s risk can differ significantly from the overall population (Scott, 2003). Risk determined by EEG, MRI and blood tests can be subjective and a poor predictor of disease development but may assist in diagnosing individual cases (Krumholz et al., 2007). An epilepsy syndrome is defined by various clinical features such as EEG, age of onset and event duration, but the cause, disease trajectory and treatment success can vary across people diagnosed with the same syndrome.

While individual neurons of epileptic patients may display unusual electrophysiological activity, seizures arise through the interaction of neurons. The current clinical definition of a seizure is simply an abnormal excessive or synchronous neuronal activity in the brain (Fisher et al., 2014). Computational models suggest that changes in neuronal arrangement, conductivity and number of connections can affect the electrophysiological behaviour of neural tissue (Demont-Guignard et al., 2009). Neural synchronisation, as measured by increased EEG activity, is also associated with cognitive function and several neurological disorders (Uhlhaas and Singer, 2006). However, there is no understanding of what sets of interactions give rise to epileptiform behaviour or how seizure patterns vary with tissue structure or complexity (Jirsa et al., 2014). It is also still unclear if any electrophysiological features are biomarkers for epileptic seizures (Demont-Guignard et al., 2009). It has been commonly assumed that only neurons are important in tissue function, with an imbalance in excitatory and inhibitory neurons playing a role in seizures (Uhlhaas and Singer, 2006), yet astrocytes and glia can also alter tissue behaviour (Cserép et al., 2021).

We will now consider epilepsy through our emergence framework. Seizures are an electrophysiological property of tissue arising from the collective interaction of neural cells. They are collaborative, not being controlled by a pacemaker cell, and dependent on the electrophysiological and chemical properties of the composing neural cells. A seizure has a range of novel features not present in individual neural cells. The definitions used to describe seizures in brain regions or networks differ from those used for single cells. For example, an important concept in investigating epilepsy is known as ‘hyper-excitability’, which is a relative term that can be applied at different scales. A single cell would be defined as being hyper-excitable if it exhibited a higher-than-normal frequency of action potentials (for its cell type). By contrast, hyper-excitability in neural networks is defined by an increase in oscillatory power in specific frequency bands in EEG recordings. Hyper-excitability in a neuron does not guarantee hyper-excitability of a neural network, and hyper-excitability in a neural network does not require hyper-excitability in the composing neurons.

Cells contributing to a seizure often display different complexity in their temporal rhythms than they would in isolation, for example, by displaying tightly synchronised (low-dimensional) electrical bursts, contrasting with the typically sparser, more random-like (high-dimensional) activity patterns outside of seizure states. Seizures may exhibit changes in their locality over time. Focal seizures, for example, start in a single area of the brain, so that the aberrant activity is localised in space. The seizure may remain localised to this area or may spread either in a single or across both hemispheres so that the aberrant activity is more spatially uniform and less localised. It is difficult or impossible to predict if a person will be epileptic, when an epileptic person will have a seizure, the form of seizure and how changes in brain structure will affect seizure type. The seizure type and its EEG response may be irreducible to the electrophysiological properties of individual cells. Some non-essential features of the emergence framework can also be assigned to seizures.

Recognising seizures as an emergent feature has important implications. It is not possible to understand and model a seizure by extrapolating from studies of single cells using a GOFR strategy, hence one of the alternative strategies needs to be adopted. Cells removed from a seizing tissue may continue to display the same properties but could also change in behaviour without interactions with other cells. Variations in tissue composition, structure and number of cells can affect seizure behaviour. While two brains may display seizures, they can have vastly different causes.

Regarding seizures as emergent can help understand many aspects of epilepsy. Changes to neuronal connectivity may be small but can lead to large shifts in neural activity. Such changes are unlikely to lead to a seizure-prone brain network when occurring in isolation. However, the combined contribution of these changes over time may cause a shift to a brain in which seizures can be readily induced. Importantly, since these individual changes may be small and time-localised, it can be difficult to predict which individual may develop epilepsy, even with knowledge of their genetic risk and epigenetic profiles (Pitkänen et al., 2016).

Treating epilepsy may then involve altering the tissue composition or structure, either through administration of anti-seizure medications, or changing neural connectivity or cellular composition. Changes in connectivity may be achieved by blocking action potential generation or propagation, altering the strength or number of synaptic connections, changing the types of connections (excitatory or inhibitory) or changing the overall excitability of the tissue (e.g., by changing pH, temperature or extracellular fluid composition). This may provide treatment options other than tissue re-sectioning and new avenues for treating uncontrolled seizures.^22^

A similar approach could be applied to understand other brain functions. This is particularly important when considering the increasing volume of neuroscience studies on ‘organoids’ and other patient-derived neural models (Nieto-Estévez and Hsieh, 2020; Pelkonen et al., 2020; Pașca et al., 2022). These tissue models provide a significant advantage from an ethical, cost and control standpoint when compared with clinical trials and other studies involving human participants (Harris et al., 2022b, 2023). However, care must be taken to ensure that the aspects around emergence discussed in this article are considered when using such models (Harris et al., 2020). In particular, the environment in which the tissue model is grown and recorded from, may play a significant role in any observed behaviour due to emergent properties arising from the interactions between the model and the environment. Conversely, tissue models, whilst simple, may provide a cornerstone for investigating emergent behaviours associated with sensory and cognitive processes in more complex neuroscience systems.

## 8 Guiding principles for emergence in neuroscience

In this article, we introduced a new framework for classifying systems and emergent behaviour whereby a system displays collective, collaborative, dependant and novel features. Novelty can be identified by a network of interacting components acquiring features that are not present in the individual components, or individual units exhibiting novel behaviour due to being part of a network. To apply this framework to neuroscientific systems in a practical sense, and with the application of appropriate strategies described in section 2, we suggest the following guiding principles:

*Multiple model systems should be used to investigate emergent behaviour.*

A system’s components, boundary and environment must be defined. Component properties in relative isolation must be identified to assess novel behaviour. The composition and arrangement of components in a system will determine what interactions are possible and their properties. Building model systems with varying composition enables determination of the most appropriate strategy for describing a system’s behaviour, what interactions are required to generate and control emergent features, the forms of novelty which arise, and limitations of simpler models for investigating a system’s behaviour. This may allow generation of new universal laws for describing system behaviour that can be used in theoretical models that bridge across multiple spatial and temporal scales. Thus, when the rules of interaction have been determined, a wider range of system structures can be created in computational models to investigate the impact on the system’s behaviour.

Theoretical models, be they physics-informed or purely data-driven, trained only on Lego-like (separable) components or a limited set of non-Lego-like (non-separable) components, their features and interaction rules, will fail to accurately describe systems of non-Lego-like components. Note that a wider array of components and systems can be created in theoretical and computational models than may be possible in physical models (e.g., an isolated synapse versus two synaptically coupled neurons). It is thus important when developing models (physical, computational or theoretical), to ensure that the component and system properties are properly validated experimentally.

In some cases, it may be difficult to create physical model systems with certain structures due to technical limitations, or due to the components self-organising into specific structures based on interaction rules between components such as minimisation of total energy. There may be geometric or energy constraints on system structures (e.g., atomic and molecular structure). There may also be size limitations, minimum number of components or energy requirements in creating/maintaining a system (e.g., cell and animal size).

*Controlling system behaviour depends on how that behaviour arises in terms of the components, their arrangement or structure/connectivity.*

Interventions on system behaviour through the lens of the GOFR strategy require changing the composing units (e.g., changing the molecules in a solution). Viewing system behaviour using the Optimistic Non-Separability strategy implies more opportunities for intervention since one can target system arrangement or structure (e.g., epileptic seizures may be modified by changing the type or number of cells, addition of a drug, altering cell connectivity or arrangement). By contrast, employing the NO-GOFR strategy implies that system behaviour can only be affected by intervening directly at the system level.

*System behaviour should be understood in a “bottom up” fashion, but recognising that system components may interact in different ways in different contexts.*

Certain isolated components or systems may not be stable, being examinable only in theory or observed in very specific contexts. In some cases, being embedded in a system may stabilise such components. However, observing the components only when within a system, risks misunderstanding the properties of the components in relative isolation. Where possible, a system should be deconstructed to assess the behaviour of isolated components to determine if the previous interactions (when part of a system) have resulted in a long-term modification to the isolated component behaviour.

As components become more complex, it often becomes more difficult to define and measure their properties, interactions, boundaries and environment. Systems may display multiple forms of emergence, making identification and classification of novelty difficult and lead to incorrect labelling. To overcome this, we must build up our understanding from the bottom up (this is not to be confused with a GOFR approach where system features are fully derivable from Lego-like components).

*Non-equilibrium system behaviour is not synonymous with emergent behaviour.*

System components are often subject to dynamic processes associated with their formation, degradation, and transition between different states. The dynamic nature of these processes can result in the rearrangement of system structures between different configurations (e.g., those associated with different local minimum energy states), which may occur over a variety of timescales. Note that the presence of the non-equilibrium behaviour does not in itself imply that the behaviour is emergent. Indeed, this behaviour may be fully derivable from knowledge of Lego-like system components. Conversely, emergence may lead to a transition from equilibrium behaviour to non-equilibrium behaviour, leading to a dynamic expression of emergent behaviour. Crucially, the dynamics themselves should not be confused with the actual form of emergence, which may correspond to a different part (potentially non-dynamic) of the system.

*Small perturbations in system composition do not necessarily result in small perturbations in system behaviour.*

It is possible for the modification of just a single component within a pre-existing system to modify or create novel features in the subsequent system, even when previous modifications have not led to such a change. For example, addition of single water molecules at a time to a cell will lead to gradual changes in intracellular water concentration and cell volume up until the cell suddenly bursts. The act of adding a single water molecule in the case in which the cell bursts is no different than the case in which the cell did not burst, however, the behaviour displayed by the system is catastrophically different. Conversely, there can be large changes in a system’s redundant components with no impact on system behaviour. Changes to non-redundant components may also not impact system behaviour if such changes are compensated for by changes to other system components (e.g., via homeostatic mechanisms).

## 9 Conclusion

In conclusion, this article provides a scientific method to identify emergent behaviour in a system; allowing us to avoid reductionism and enabling us to identify and categorise different forms of emergent behaviours relevant to neuroscience. It may aid in designing new tools, experimental methods and systems for investigating and identifying emergent behaviour. It may also allow for the further development of an explanatory strategy that has received less attention than others, ‘Optimistic Non-Separability’. Optimistic Non-Separability provides a sort of “middle ground” for thinking about emergence. In particular, ONS points to the idea that we may need to study neuronal behaviour in a variety of contexts and environments, including both natural and artificial ones.

## Data Availability

The original contributions presented in the study are included in the article/supplementary material, further inquiries can be directed to the corresponding author.

## Footnotes

1 GOFR is a playful nod toward the characterisation of classical approaches to artificial intelligence as “Good Old-Fashioned Artificial Intelligence (GOFAI)” (Haugeland, 1985). In a similar manner to our use of the term GOFR, the term GOFAI was not meant pejoratively, but was instead intended to capture a core set of ideas that animated early research in artificial intelligence.

2 We will use the term “level” in a variety of ways, as is common in the literature. These include levels of observation, levels of description, levels of organisations, levels of reality, etc. Often, levels are tied to concepts of scale, for example, contrasting between microscopic and macroscopic structures and/or dynamics. In other uses, levels may refer to specific types of components, such as individual neurons or brain regions.

3 We distinguish between a relatively isolated component disregarding any environment and one within in an empty environment. Placing a component into an empty environment can be considered a form of interaction which does not change the components properties but does give it new spatiotemporal properties.

4 Note that, rather than model all system components, it may be possible to create simplified models involving statistical distributions of component properties or states. Such approaches are often used, for example, in machine learning or more general data-driven methods.

5 Since there were four different strategies associated with accepting Derivability (GOFR, Practical Pessimism, Optimistic Non-Separability, Pessimistic Non-Separability), are there four corresponding strategies associated with rejecting Derivability? Could we reject Derivability while maintaining Practical Optimism and/or Separability? Since Derivability involves a general claim regarding every system-level brain function, it is of course possible to reject it only partially: we might hold that some system-level brain functions are derivable in the manner described above while others are not. Technically, this is still a rejection of Derivability – and in fact it is perhaps the most likely form a rejection would take (since the claim that no system-level brain function can be derived from the characteristics of system components is quite extreme). In the case of partial rejection, one could adopt restricted versions of the four strategies (GOFR, Practical Pessimism, ONS and PNS) toward the cases where Derivability is accepted. However, in cases where Derivability is rejected, Practical Optimism and Separability must also be rejected since they both effectively describe further conditions on Derivability.

6 The proposed defunding of cognitive neuroscience within the Human Brain Project led to the signing of an open letter threatening to boycott the project by over 350 members of the neuroscience community. Ultimately, this led to a significant governance restructure.

7 The distinction between ontological and epistemological forms of emergence is closely related to the distinction between strong and weak forms of emergence. However, there are important differences between these two distinctions. See Bedau (2008) for discussion.

8 We remark that whilst supervenience can be thought of as a necessary condition for emergence, there are strong arguments against its usefulness (Kim, 2006). Whilst we will consider supervenience as a concept in the examples we discuss, we will focus primarily on other aspects of emergence.

9 Such phenomena can be understood using the GOFR strategy upon reframing to incorporate such ‘sideways’ interactions.

10 Note that, like emergence, the concept of complexity is also a tangled one, with a variety of possible interpretations. See Ladyman et al. (2013) for an attempt to clarify the concept of complexity. They also argue for a conceptual separation between complexity and emergence, although on different grounds.

11 These models also often (but not always) require the variables of interest to be of a similar type between the lower- and higher-levels to enable explicit calculations.

12 On this subject, Huneman and Humphreys write: “If we can pick out some properties or processes as emergent according to some computational criteria applied to a simulation of the matter of facts they belong to, and if those criteria are not dependent upon our cognitive abilities but objective in the sense that mathematical properties might be said to be objective, is this status of “being emergent” ipso facto an ontological property, e.g., a property which concerns not only our concepts for describing matters of fact, but this matter of fact itself?” (Huneman and Humphreys, 2008).

13 In the degrees of freedom (DoF) framework, emergence can be understood as the elimination of DoF in characteristic states of the emergent features with respect to those of the component properties. Elimination here implies that certain properties and behaviour of the emergent features are independent of the eliminated DoF. This notion differs from that of reduction, in which certain DoF are fixed at some constant value; and from restriction, whereby DoF are allowed only to evolve in a restricted space (Wilson, 2010). Importantly, the eliminated DoF should pertain only to the properties of the system components. The DoF perspective is consistent with the Optimistic Non-Separability strategy since it implies that the system components obey distinct rules when composed together, which may be different depending on the system composition. The DoF approach is a similar, but stronger, emergence condition to that in the information-based closure approaches, with the latter only requiring DoF elimination in the space of information, rather than in the underlying system.

14 Fusion, which rejects supervenience between levels, postulates that the individual components in an emergent system lose certain properties or identities when *fused*. Humphreys argues that fusion is a “real physical operation, not a mathematical or logical operation on predicative representations of properties.” (Humphreys, 1997). This would imply that the individual neurons could no longer be regarded as distinct entities when operating in a network. In a less extreme example, two cells may interact, forming a novel system, this may result in downregulation of some proteins or loss of entire organelles within the cells. The cells are still present in the combined system, but they have lost certain properties. The specific fusion events may enable classification and detection of emergence occurring but are unlikely to provide any information on the properties of the emergent phenomena. Since the fusion perspective rejects Derivability, it is an example of a NO-GOFR strategy.

15 When units are removed from a system, they can retain behaviours associated with their previous interactions, and not behave as the original, isolated unit. It is also possible to have an emergent feature arise in one system property (e.g., electrophysiology) but not in others (e.g., total mass).

16 In the context of social networks, emergence is often reported in the dynamics of opinion formation (Antonopoulos and Shang, 2018; Evans and Fu, 2018). As such, one could go so far as to think of entire brains as representing lower-level systems embedded in a social network.

17 Under/overpacking density may also lead to interaction frustration, which was originally developed to describe conflicting arrangements of many particle systems leading to disorder.

18 From an epistemological perspective, one can develop ‘physical’ models already at macroscopic scales (Freeman, 2000; Breakspear, 2017) or derive mean-field models that concentrate on low dimension approximations to larger scale models (Deco et al., 2008). From a practical standpoint, these macro-scale models might be better suited to the current data-collection capabilities than scaled-up microscopic models, since our abilities to resolve simultaneous spiking activity in large networks remain limited. From a theoretical perspective, the increased tractability of macro-scale models makes them amenable to dynamical systems analysis that can identify core dynamical states underpinning cognitive and sensory processes. For example, this larger scale perspective facilitates exploration of the “critical brain hypothesis” that posits that brains self-organise to operate close to (but before) a phase transition between distinct dynamical regimes (Wilting and Priesemann, 2019). Discrepancies between macro-scale predictions and observed phenomena could then provide useful information on missing features of micro-scale models, for example, highlighting the importance of spike time dependent plasticity, suggesting a way to bridge between scales. A more contemporary perspective attempts to use the same dynamical systems concepts from a more data-driven approach by identifying low dimensional descriptions (the so called “neural manifolds”) of high-dimensional data to uncover how groups of neurons or populations collectively encode information (Perich et al., 2025). Given the rapid development of new machine learning tools, coupled with advancements in recording technology, understanding the link between emergence in data-driven models and that in more mechanistic models is a potential avenue for future research.

19 This does not refer to the complexity of the system components and interactions. Those types of complexity are included in Table 4 among other features which we regard as non-essential for emergence.

20 In highly ordered crystal structures, this could be thought of as the structure switching from isotropic to anisotropic.

21 Novel features are often used as biomarkers in biomedical research, predicting clinical outcomes assessments and patient symptoms or defining the state of a system (e.g., the pluripotency of stem cells). This requires that the biomarkers in each system can be related to the behaviour being assessed in a patient. The appropriate use of these types of biomarkers relies on the accuracy in their ability to predict behaviour in the complex system (patient), their robustness across different systems (patients), and their uniqueness in relation to the clinical behaviour being assessed (Harris et al., 2024).

22 For instance, a phase 1/2 clinical trial involving implanting IPSC-derived inhibitory neurons into seizure focal zones of treatment refractory patients has shown significant reduction in seizures (NCT05135091).
